# Influence of the spatial confinement on the self-focusing of ultrashort pulses in hollow-core fibers

**DOI:** 10.1038/s41598-019-45940-3

**Published:** 2019-07-02

**Authors:** Aurora Crego, Enrique Conejero Jarque, Julio San Roman

**Affiliations:** Grupo de Investigación en Aplicaciones del Láser y Fotónica, Departamento de Física Aplicada, University of Salamanca, Salamanca, E-37008 Spain

**Keywords:** Ultrafast photonics, Nonlinear optics

## Abstract

The collapse of a laser beam propagating inside a hollow-core fiber is investigated by numerically solving different nonlinear propagation models. We have identified that the fiber confinement favors the spatial collapse, especially in case of pulses with the input peak power close to the critical value. We have also observed that when using pulses in the femtosecond range, the temporal dynamics plays an important role, activating the spatial collapse even for pulses with input peak powers below the critical value. The complex self-focusing dynamics observed in the region below the critical power depends on the temporal evolution of the pulse and, also, on the interaction between the different spatial modes of the hollow-core fiber.

## Introduction

One of the first nonlinear effects that appears when a laser beam propagates through a medium is the variation of the refractive index with the intensity of the beam: the optical Kerr effect. The immediate consequence of this intensity dependent refractive index is the self-focusing of the beam towards the highest intensity regions, usually its central part. It is well-known that for input peak powers above a threshold value, called critical power, the nonlinear propagation theory predicts that the beam will undergo spatial collapse.

This self-focusing process is very relevant in many different contexts such as in the formation of the Townes soliton^1–3^, in the design of laser resonators to avoid the appearance of hot spots (local self-focusing processes)^4^, as a natural limit in the intensity up-scaling of fiber amplifiers^5^, as the first step of the filament formation^6^, as a limit in some post-compression schemes^7^ or in the material processing context^8^, where the self-focusing process triggers other higher nonlinear effects that worsen the output result.

The full understanding of the self-focusing process implies the determination of the minimum peak power value needed to activate the self-focusing dynamics, called the critical power. This fundamental parameter was identified already in the early self-focusing studies^1^ and, since then, its definition has been revisited many times due to its dependence with the spatial profile^9,10^, the temporal and non-paraxial dynamics^11,12^, the focusing geometry^13^, the spatial impurities^14^, the presence of the Raman effect^15^, etcetera. All these dependencies are an evidence of the rich and complex interaction between self-focusing and other linear and nonlinear effects, which is the reason why it is still an active research topic. Moreover, once the critical power is defined in a particular context, a formula to estimate the collapse distance has often been proposed. For the case of a free propagating beam we have the well-known Marburger’s formula^16,17^ and similar formulas developed in other studies^10,18,19^.

The self-focusing process in guiding systems (optical fibers, photonic crystal fibers and hollow-core fibers (HCFs)) has been theoretically studied in the past obtaining contradictory conclusions. Tempea and Brabec^20^ found that the critical power of beams propagating in HCFs is substantially higher (5 times) than if they propagate in free space. Later, Fibich and Gaeta obtained basically the opposite result, a critical power slightly lower for the fundamental mode of the HCF than for a free Gaussian beam^9^. Similarly, Farrow and coworkers observed stationary solutions in a fiber amplifier for peak powers below the critical power of a free Gaussian beam, suggesting a decrease of the real critical power in the fiber, although concluded that the critical power in a step-index fiber was nearly the same as in free space^5^. Finally, self-focusing below the critical power of a free Gaussian beam was also observed in photonic crystal fibers^21^, suggesting again a different behavior between a free and a spatially confined propagation.

In this work we will study the self-focusing dynamics of a beam propagating in HCFs, which is the most usual technique to achieve few-cycle laser pulses in the near infrared^22,23^. The standard post-compression process requires the spectral broadening of the pulse during its nonlinear propagation inside a gas filled HCF and its subsequent spectral phase compensation to finally obtain the shortest possible output. The activation of high nonlinear effects, such as ionization, which could occur if the beam self-focuses inside the HCF, deteriorates the output results, being therefore a limitation to up-scale the standard post-compression set-ups^7^. We will try to gain some insight into this problem comparing the self-focusing dynamics of free beams and spatially confined beams (beams propagating in a HCF) using two different theoretical models based on the nonlinear Schrödinger equation. We have verified, with both models, that the collapse distance of the fundamental mode inside the HCF is appreciably different than in free space due to the spatial confinement, specially for peak powers slightly greater than the critical power of the fundamental mode of the HCF (${P}_{cr}=1.86225{\lambda }^{2}$/$(4\pi {n}_{0}{n}_{2})$)^9^, that we used as the reference value during the whole work. Moreover, the time-dependent model shows that the spatial collapse can appear also for pulses with peak power below *P*_*cr*_, being the temporal pulse evolution and the interference between different spatial modes the two main ingredients of this complex dynamics. In the last part of this work we discuss how the self-focusing dynamics, accompanied by other high order nonlinear terms, would manifest in a real experiment, demonstrating that pure self-focusing studies help to identify the energy limit of the HCF post-compression scheme.

## Results and Discussion

To study the collapse process in HCFs we have developed two numerical models (see the Methods section). The first model focuses on the spatial dynamics ((1 + 1)D model). It includes the diffraction and the self-focusing effects, not taking into account any temporal distortion of the pulse. This is a standard model to simulate the collapse dynamics of a laser beam^9,24^ but, as we want to study the self-focusing process in the post-compression context, we need to verify the possible influence of the evolution of the temporal structure of the pulse, which could be relevant for pulses in the femtosecond regime. For this reason we have developed a second model that includes the complete spatio-temporal dynamics of a laser pulse in a HCF ((2 + 1)D model). This second model includes the diffraction and self-focusing effects together with the dispersion, self-phase modulation and self-steepening. In the last part of this work, we add the ionization of the medium to the (2 + 1)D model in order to see how self-focusing affects the general dynamics in a more realistic model.

One of the main issues when numerically studying the self-focusing process is the collapse criterion, which determines the propagating distance at which the spatial collapse takes place. Looking into the literature, one finds that to visualize the self-focusing dynamics some authors use the beam spatial width evolution^5,25^ while others use the field amplitude or intensity evolution^9,14^, but to define a collapse criterion most of them use a field amplitude threshold. This type of field amplitude criterion is not adequate for our system for two reasons: first because we are dealing with beams propagating in HCFs, whose modes are intrinsically leaky. The relevant absorption losses present in HCF would affect those spatial collapses occurring at short distances differently than those occurring at long distances, although both beams would present similar spatial widths. The second reason is that we want to include the evolution of the temporal structure in this self-focusing study. We will see soon that there is strong self-compression dynamics during the nonlinear propagation that affects the peak intensity evolution. Therefore, to isolate the spatial dynamics from other terms (absorption or temporal dynamics), we define the spatial collapse, for both simulation models, when the spatial width, measured as the half width at half maximum (HWHM) from the peak intensity, drops below 0.1 times the initial beam waist (*w*_0_ = 110 *μ*m for the fundamental mode of a 150 *μ*m core radius HCF). Using this purely spatial collapse criterion we find similar collapse distances for a free Gaussian beam than those obtained with Fibich’s formula (Eq. 12 in^10^). A different collapse criterion could change the quantitative results, the collapse distances, but qualitative results, as how the spatial confinement induced by the HCF affects the collapse dynamics, would remain the same.

### The time-independent model ((1 + 1)D model)

**Figure 1:**
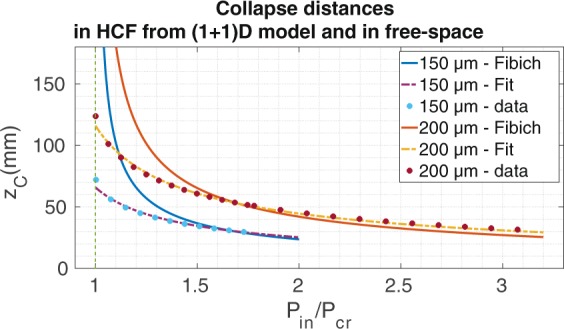
We show the collapse distances of the *EH*_11_ mode at 800 nm as a function of the input peak power and of the core radius of the HCF. Circles represents the collapse distances obtained from the (1 + 1)D numerical model for HCFs of 150 *μ*m and 200 *μ*m core radius, dashed lines are obtained from the estimation formula (Eq. 2) and solid lines correspond to the prediction from the free-space Fibich’s formula^10^. *P*_*cr*_ is the critical power for the fundamental mode of the HCF as defined in^9^.

To show how the self-focusing dynamics is affected by the spatial confinement induced by the fiber we have simulated the nonlinear propagation of the fundamental mode of a HCF, the *EH*_11_ mode, centered at 800 nm. The initial condition used to solve numerically Eq. 3 (see Methods) is, therefore:

1 $$E(r,z=0)\propto \{\begin{array}{ll}{J}_{0}({u}_{0}r/{r}_{F}) & r\le {r}_{F}\\ 0 & r > {r}_{F}\end{array},$$

where *u*_0_ and *r*_*F*_ are the first zero of the Bessel *J*_0_ function and the fiber core radius, respectively. We will compare the collapse distances of the *EH*_11_ mode propagating inside a HCF obtained numerically, with the free-space collapse distances predicted by the Fibich’s formula (Eq. 12 in^10^). To apply the formula we use the waist of the Gaussian that best fits the fundamental mode of the HCF (${w}_{0}=0.65{r}_{F}$) and the critical power corresponding to the *EH*_11_ mode^9^. Figure 1 shows the numerical collapse distances (circles) and the prediction obtained through Fibich’s formula (lines) for different input pulse energies and fiber core radius. We obtain two important conclusions: first, we do not observe spatial collapse for peak powers below *P*_*cr*_. Second, and more important, the tendency of the collapse distances in the HCF is different from those obtained from the free-space propagation, especially when the input peak power is close to the critical power $({P}_{in}\gtrsim {P}_{cr})$. The collapse distances are shorter in the HCF than in free space and, surprisingly, the collapse distances in the HCF, instead of diverging when getting close to the threshold value (*P*_*cr*_) as occurs in the free-space propagation case, disappear abruptly at the critical power. This new behavior is related to the reduction of diffraction and to the losses induced by the spatial confinement of the HCF. Note that at high input peak powers, where the self-focusing term dominates against the absorption and the diffraction, the difference between the collapse distances in the HCF and in free space vanishes. The conclusion obtained from the (1 + 1)D model is clear: the spatial confinement of the beam favors the self-focusing dynamics, specially for peak powers close to the critical power.

Fitting our numerical results we can obtain a simple formula to predict the collapse inside the HCF:

2 $$\frac{{z}_{c}}{{z}_{R}}=(\frac{0.625}{{(p-0.715)}^{0.6346}})$$

where $p={P}_{in}$/${P}_{cr}$, being *P*_*cr*_ the critical power of the fundamental mode of the HCF^9^, and *z*_*R*_ is the Rayleigh length ${z}_{R}=\pi {w}_{0}^{2}$/*λ*. We have plotted the results obtained from Eq. 2 as coloured dashed lines in Fig. 1 to show the agreement between numerical and analytical results. Only values with ${P}_{in}$/${P}_{cr}\ge 1$ have been plotted as we have not observed collapse for input peak powers below *P*_*cr*_ with this (1 + 1)D model.

### The time-dependent model ((2 + 1)D)

**Figure 2:**
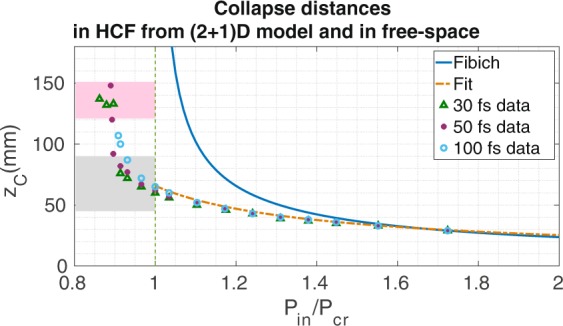
We show the collapse distances of the *EH*_11_ mode at 800 nm as a function of the input power and duration of the pulse for a HCF with core radius 150 *μ*m. The different markers are the collapse distances obtained from numerical (2 + 1)D simulations for different laser pulse duration, the solid line represents the collapse distances predicted by Fibich’s formula in free space, while the dashed line corresponds to the fit obtained from the (1 + 1)D model (Eq. 2).

A more complete description of the pulse propagation in the HCF is simulated with a time-dependent model ((2 + 1)D model) that includes spatial and temporal dynamics (Eq. 6 of the Methods). The temporal effects included in this model are dispersion, self-phase modulation and self-steepening. As it is usual in any self-focusing study^12,24,25^, and trying to isolate this spatial nonlinear process, neither the effect of the plasma induced by the pulse nor the losses related to the ionization process or the presence of the plasma are included, as they would partially prevent the self-focusing process. The collapse distances obtained with the (2 + 1)D numerical model for different pulses, centered at 800 nm, coupled initially to the *EH*_11_ mode of a HCF with 150 *μ*m core radius and filled with 1 bar of Ar, are presented in Fig. 2. As a reference, we have also plotted the Fibich’s formula for free-space propagation^10^, (solid line), and our new fit (Eq. 2) with an orange dashed line.

Two different regions can be observed in Fig. 2: the high peak power region (HPR, ${P}_{in} > {P}_{cr}$) and the new low peak power region (LPR, ${P}_{in} < {P}_{cr}$), where the (2 + 1)D numerical model presents spatial collapses. In the HPR the two numerical models present basically the same tendencies, which demonstrates that in this regime the self-focusing process weakly depends on the input pulse duration. All the collapse distances in this regime are well described by the (1 + 1)D model represented by Eq. 2. For these cases self-focusing overcomes diffraction, absorption, and also all the temporal effects, dominating the propagation of the beam.

#### Role of the spatial modes in the self-focusing dynamics: a multimode self-compression

The collapse dynamics observed in the LPR is much more rich and complex. First we should recall that there are already several references that present self-focusing for peak powers below the critical value^5,21,25^, all of them related with multimode guiding systems. HCFs are multimode systems and it is not surprising that their multimode nature could be relevant to understand the self-focusing dynamics observed. Although we assume that at the capillary entrance the beam is purely coupled into the fundamental mode of the HCF, the nonlinear propagation through the HCF induces an important energy transfer towards higher spatial modes. These modes, besides presenting a narrower spatial distribution and activating the self-focusing process, have an anomalous dispersion response, which means that they could temporally self-compress. To understand the rich spatio-temporal dynamics that one could expect from this new (2 + 1)D model, we present in Table 1 the values of the group velocity and the group velocity dispersion (GVD) at 800 nm for the first four spatial modes of a HCF with 150 *μ*m core radius and filled with argon at a pressure of 1 bar.

**Table 1:** Group velocity and GVD values for the fundamental and first three excited modes at 800 nm in a HCF with 150 *μ*m core radius and filled with argon at 1 bar.

*λ* = 800 nm	*EH*_11_	*EH*_12_	*EH*_13_	*EH*_14_
*v*_*g*_ (nm/fs)	299.916	299.913	299.908	299.901
GVD (*fs*^2^/m)	34.56	−9.40	−35.86	−101.23

**Figure 3:**
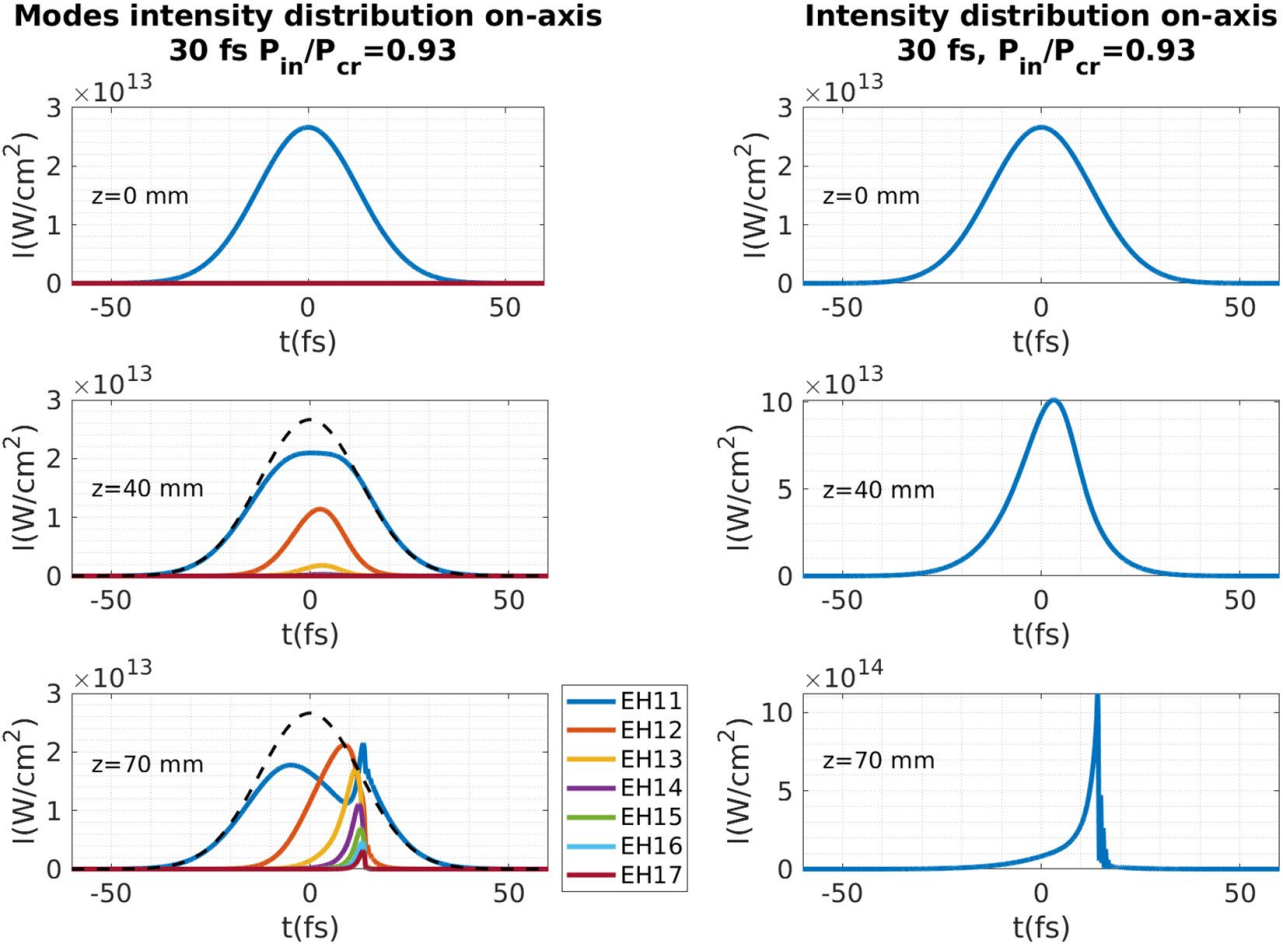
Left column corresponds to the on-axis temporal intensity distribution of different modes and right column corresponds to the on-axis temporal intensity distribution of a 30 fs pulse at 800 nm with ${P}_{in}=0.93{P}_{cr}$, propagating in a 150 *μ*m core radius HCF filled with 1 bar of Ar for three propagation distances. The left column includes the intensity distribution of the fundamental mode at z = 0 with a dashed line for comparison.

Figure 3 shows the pulse intensity distribution of a 30 fs pulse with ${P}_{in}=0.93{P}_{cr}$ at 800 nm (right column) and the intensity distribution of the first seven spatial modes (left column), at three propagation distances. At the beginning only the fundamental mode contributes (Fig. 3 top left), but as the pulse propagates through the HCF there is an energy transfer to higher spatial modes that mainly occurs in the most intense part of the pulse (Fig. 3 middle left). The new generated spatial modes send energy back to the fundamental mode during their propagation, inducing an interference in the fundamental mode (Fig. 3 bottom left). Close to the collapse distances (72 mm for this case), almost all the modes contribute in the trailing part of the pulse simultaneously generating a high interference peak, as can be seen in the evolution of the on-axis temporal intensity distribution of the pulse (Fig. 3 bottom right). This figure demonstrates how the particular nonlinear mixture of the different spatial modes distorts the temporal intensity distribution in such a way that it shows an unexpected self-compression process, and a subsequent increase of the peak intensity.

#### Self-focusing dynamics in the low power and high power regimes

Regarding the spatial dynamics, the presence of higher spatial modes also induces oscillations in the beam spatial width, as shown in^25^, that could eventually produce the spatial collapse of the beam. This complex spatio-temporal nonlinear evolution explains the collapses in the LPR shown in Fig. 2 and the influence of the temporal pulse duration. Moreover, the tendency of the collapse distance in the LPR is completely different than in the HPR. While in the HPR we have obtained a quite smooth tendency, increasing the collapse distance continuously when getting close to the critical power, the LPR presents a discrete tendency (specially visible for the shortest pulses), showing collapses for some particular distance regions noted as shadowed rectangles in Fig. 2.

**Figure 4:**
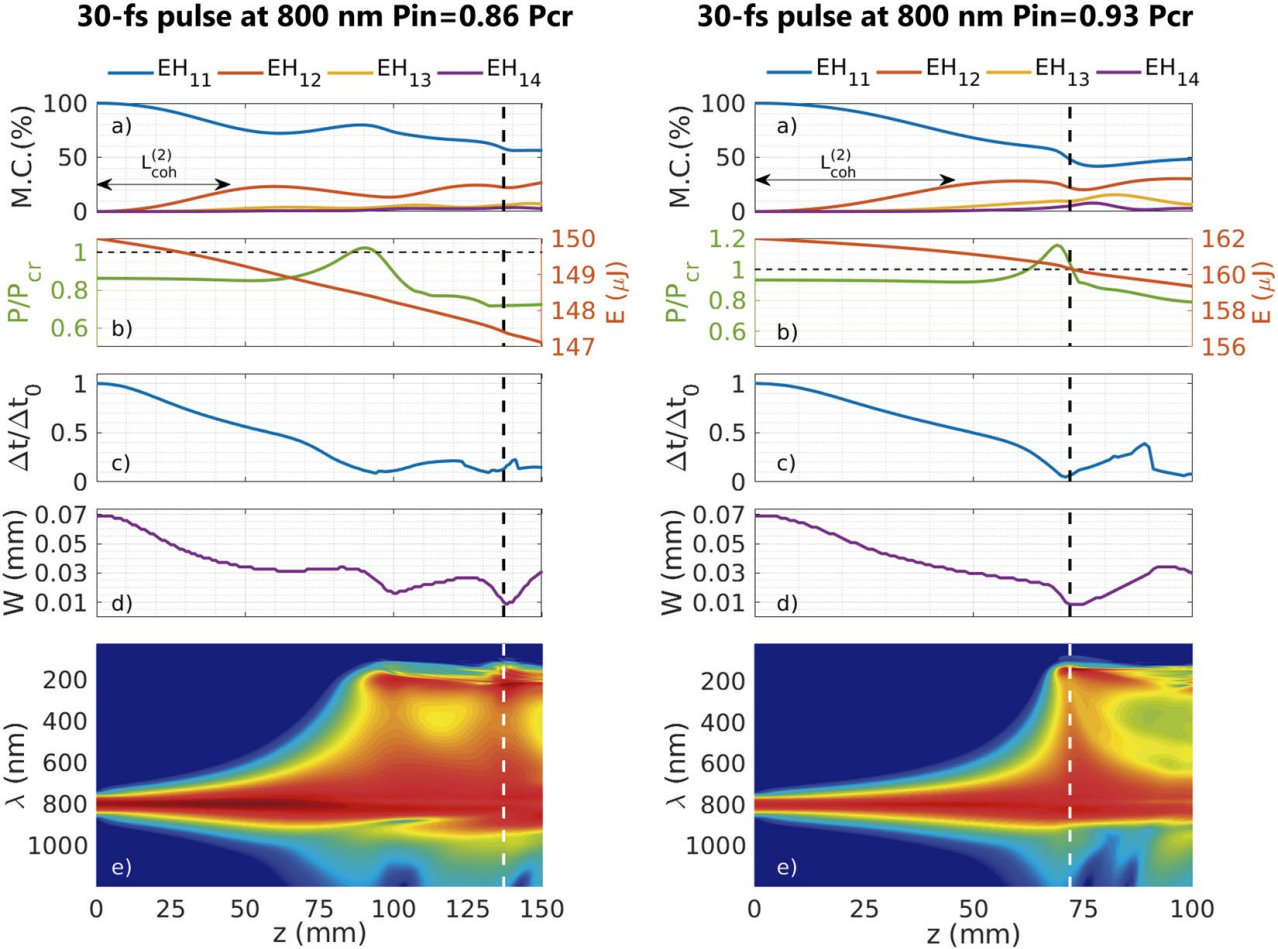
Left (right) column corresponds to the propagation of a 30 fs pulse at 800 nm, with 150 *μ*J input energy and ${P}_{in}=0.86{P}_{cr}$ (162 *μ*J input energy and ${P}_{in}=0.93{P}_{cr}$), propagating in a 150 *μ*m core radius HCF filled with 1 bar of Ar. Each column shows the evolution of the percentage mode contribution (M.C.) of the first four spatial modes (**a**), the evolution of the peak power and the pulse energy, indicating the *P*_*cr*_ threshold with an horizontal dashed line, (**b**), the evolution of the on-axis pulse duration (**c**), the evolution of the spatial width (**d**) and the evolution of the on-axis spectrum (log scale) (**e**) with the propagation distance. A vertical dashed line indicates the collapse distance for each case 137 mm (72 mm). The minimal pulse duration obtained would be 2.7 fs (left) and 1.5 fs (right). Note the different length of the z-coordinate for each case.

To unveil the origin of these collapse tendencies in the LPR we show in Fig. 4 a complete set of plots for two different collapses that summarizes the observed phenomenology. Each column of Fig. 4 corresponds to the propagation of a 30 fs pulse at 800 nm, with 150 *μ*J (${P}_{in}=0.86{P}_{cr}$, left) and 162 *μ*J (${P}_{in}=0.93{P}_{cr}$, right) of input energy, propagating in a 150 *μ*m core radius HCF filled with 1 bar of Ar. Each column shows the evolution of the percentage contribution of the first four spatial modes (a), the evolution of the peak power and the pulse energy (b), of the on-axis pulse duration (c), of the spatial width (d) and of the on-axis spectrum (in log scale) (e), with the propagation distance. The vertical dashed lines indicate the collapse distance for each case (137 mm (left), 72 m (right)). Figure 4(a) only shows the contribution of the first four spatial modes for clarity, although we use 30 spatial modes in the simulations.

The first important thing to note is the self-compression process commented before, and shown in Fig. 4(c), which induces an increase of the peak power (shown in Fig. 4(b)). In fact, the collapse distance always occurs after the peak power has surpassed *P*_*cr*_ during the propagation inside the HCF, as one would expect. The self-compression dynamics presents some features of a standard solitonic self-compression (see^26,27^), although the process here is more complicated. As in a solitonic process, the self-compression is accompanied by a dispersive wave generation (DWG) (see Fig. 4(c,e)), that occurs simultaneously with the spatial collapse, as can be seen in Fig. 4(d,e). The complex nonlinear coupling between the spatial and the temporal dynamics is also corroborated by the evolution of the population of the different spatial modes forming the pulse (see Fig. 4(a)). The last important thing to note is the oscillatory nature of the spatial collapse (Fig. 4(c)) in contrast with the free-space monotonous catastrophic collapse, in agreement with other self-focusing studies in multimode confined media^25^.

Regarding the oscillatory nature of the collapse, it is well-known that the transfer of energy between two spatial modes shows an oscillatory behavior related to a characteristic coherence length, ${L}_{coh}=\pi$/$({\beta }_{1}-{\beta }_{2})$, where *β*_1_ and *β*_2_ represent the propagation coefficient of each mode^20^. In our case the pulse is composed by a mixture of several modes, each of them with its own propagation coefficient, which makes this oscillatory behavior more difficult to identify. Nevertheless, we can use these coherence lengths to remark once more the multimode nature of the self-focusing dynamics in the LPR. In the HCF one can define the coherence length between the fundamental and the *EH*_1*m*_ mode as ${L}_{coh}^{(m)}=\pi$/$({\beta }^{(1)}({\omega }_{0})-{\beta }^{(m)}({\omega }_{0}))$, where ${\beta }^{(n)}({\omega }_{0})$ represents the propagation coefficient of the *n*th-spatial mode in the HCF ($n=1$ representing the fundamental mode) at the central frequency ${\omega }_{0}$. At ${L}_{coh}^{(2)}$, which in the parameters of Fig. 4(a) is 45 mm, the energy transferred from the fundamental to the 2^*nd*^-mode should be maximum, for a pure two-mode system. Figure 4(a) shows that we are not exactly in this pure case because the maximum transfer of energy to the second spatial mode appears at slightly longer distances.

Assuming ${L}_{coh}^{(2)}$ as a good estimation, one can expect that from that distance on, a decrease of the spatial width of the beam should start to happen, as is obtained (see Fig. 4(d)). From ${L}_{coh}^{(2)}$ two different scenarios could appear depending on the input peak power: one in which the self-focusing process is arrested by other terms (diffraction, absorption and dispersion), as occurs in Fig. 4 (left). In this case the pulse reaches $2{L}_{coh}^{(2)}$, where the energy transferred to the second spatial mode is returned back to the fundamental and, therefore, the spatial collapse cannot occur yet. The other scenario happens when the self-focusing dominates the evolution before reaching $2{L}_{coh}^{(2)}$, and the spatial collapse takes place between ${L}_{coh}^{(2)}$ and $2{L}_{coh}^{(2)}$. This spatial collapse, shown in Fig. 4 (right), is accompanied by an important self-compression process and the subsequent generation of a dispersive wave (DWG), all these effects occurring almost simultaneously.

The first scenario commented above, which does not show a spatial collapse between ${L}_{coh}^{(2)}$ and $2{L}_{coh}^{(2)}$, could show it in a second coherence cycle when the energy transfer goes again from the fundamental to the second spatial mode. This second collapse process should occur between $3{L}_{coh}^{(2)}$ and $4{L}_{coh}^{(2)}$ (135 mm and 180 mm). A spatial collapse in a second period is exactly what occurs in Fig. 4 (left), linked again to a second DWG process, but the collapse distance does not coincide well with the estimation obtained from the coherence length ${L}_{coh}^{(2)}$. To obtain a better estimation we have to take into account not only the second mode, but also the third mode. In that case, following the same reasoning used before, one estimates that the pulse would have a second chance to collapse between $2{L}_{coh}^{(2)}+({L}_{coh}^{(2)}+{L}_{coh}^{(3)})$/2 and $2{L}_{coh}^{(2)}+({L}_{coh}^{(2)}+{L}_{coh}^{(3)})$, which in our case is between the propagation distances 121 and 152 mm, as observed in Fig. 4 (left). This oscillatory dynamics, related to the periodic energy transfer between the different spatial modes, demonstrates the multimode nature of the self-focusing process and is the reason why we observe discrete collapse distance regions.

To better visualize this discrete behavior we have indicated in Fig. 2 these two regions in which the spatial collapse could take place: the grey and pink shadowed rectangles, are defined as the regions between ${L}_{coh}^{(2)}$ and $2{L}_{coh}^{(2)}$ (grey area), and between $2{L}_{coh}^{(2)}+({L}_{coh}^{(2)}+{L}_{coh}^{(3)})$/2 and $2{L}_{coh}^{(2)}+({L}_{coh}^{(2)}+{L}_{coh}^{(3)})$ (pink area). Although the spatial beam collapse is driven by more than three spatial modes, as suggested by the limits commented above, these regions estimate quite well the collapse distance limits, specially for the shortest pulses.

The behaviour in the HPR region is less rich in the sense that the self-focusing dominates the rest of spatio-temporal effects that are included in the (2 + 1)D model. We have observed, for all the different pulse durations that we used, a very similar dynamics to that presented in the right column of Fig. 4 (the higher input peak power case). Indeed, the higher input peak power cases show such a strong self-focusing process, leading to a short collapse distance, that the spectral broadening is not strong enough to activate the DWG and, as a consequence, the minimal pulse duration is longer than those obtained in the LPR. This is the main difference between the self-focusing dynamics in the LPR and in the HPR.

#### Influence of the parameters of the input laser pulse and the filling gas on the self-focusing dynamics

**Figure 5:**
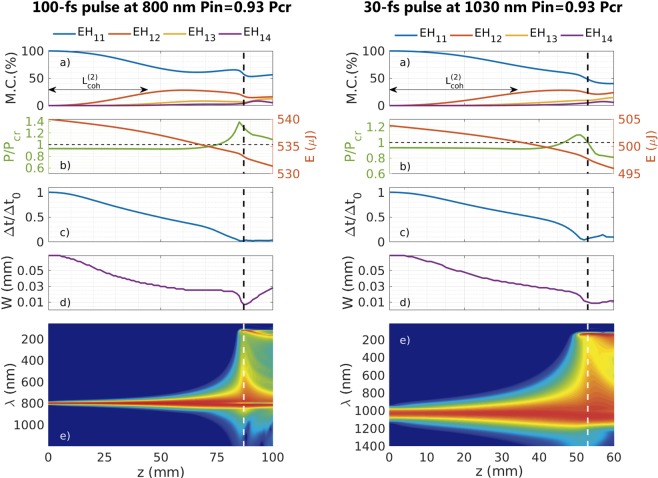
Left column corresponds to the propagation of a 100 fs pulse at 800 nm with ${P}_{in}=0.93{P}_{cr}$, propagating in a 150 *μ*m core radius HCF filled with 1 bar of Ar. Right column corresponds to the propagation of a 30 fs pulse at 1030 nm with ${P}_{in}=0.93{P}_{cr}$, propagating in the same HCF filled with 1 bar of Ar. Each column shows the evolution of the percentage mode contribution (M.C.) of the first four spatial modes (**a**), the evolution of the peak power and the pulse energy, indicating the *P*_*cr*_ threshold with an horizontal dashed line, (**b**), the evolution of the on-axis pulse duration (**c**), the evolution of the spatial width (**d**) and the evolution of the on-axis spectrum (log scale) (**e**) with the propagation distance. A vertical dashed line indicates the collapse distance for each case: 87 mm for the 100-fs pulse at 800 nm and 53 mm for the 30-fs pulse at 1030 nm. The minimal pulse duration would be 1.9 fs (left) and 1.2 fs (right). Note the different length of the z-coordinate for each case.

It is interesting to analyze how the properties of the laser pulse and the gas inside the HCF affect the self-focusing dynamics we have presented. Regarding the effect of longer input pulses, we show in Fig. 5 (left) the dynamics of a 100-fs pulse with *P*_*in*_/${P}_{cr}=0.93$ (which can be compared with the same input peak power case for a 30-fs pulse shown in Fig. 4 (right)). As observed, the main difference here is that the spatial collapse takes place at a longer distance for the 100-fs pulse, but showing very similar spatio-temporal dynamics.

Another important parameter is the central wavelength of the laser used. For instance, ultrashort ytterbium-based laser systems have gained a lot of interest in recent years due to their high average power levels which make them useful in scientific as well as industrial applications, and the compression of Yb-based pulses is a current hot topic in the field (see, for instance^28–33^). We have performed simulations for the wavelength of an Yb-doped source, 1030 nm. In particular, we have studied the propagation of a 30 fs laser pulse, centered at 1030 nm, with *P*_*in*_/${P}_{cr}=0.93$ through a HCF with 150 *μ*m core radius HCF filled with 1 bar of Ar (we have used the nonlinear refractive index *n*_2_ value given in^29^).

**Figure 6:**
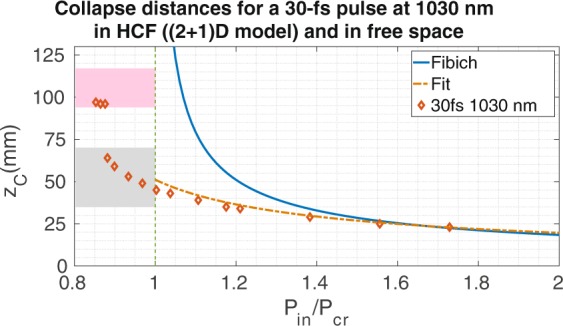
We represent the collapse distances of the *EH*_11_ mode as a function of the input power for a HCF with core radius 150 *μ*m. The markers are the collapse distances obtained from (2 + 1)D simulations for a 30-fs pulse at 1030 nm, the solid line shows the collapse distances predicted by Fibich’s formula in free space and the dashed line corresponds to the fit obtained from the (1 + 1)D model (Eq. 2). The gray and pink areas for these parameters go from 35 to 70 mm and from 94 to 117 mm, respectively.

Taking into account that the anomalous dispersion response of the gas increases with the wavelength^34^, as shown in Table 2, one expects to observe a stronger self-compression process for the Yb-doped than for the Ti:Sa laser and, therefore, an earlier spatial collapse. This is exactly what can be observed when comparing Fig. 5 (right) and 4 (right). Except for the spatial collapse position, the general self-focusing dynamics is very similar for both wavelengths. Indeed we have done a series of calculations for the Yb-doped fiber laser to see if we find the same collapse distance trend obtained for the Ti:Sa laser. Figure 6 shows again that the self-focusing dynamics are very similar and, moreover, our fit equation (Eq. 2), adapting the *P*_*cr*_ for this case, provides an excellent agreement. We have also plotted the gray and pink areas obtained from the coherent length estimation for this situation, recovering again a good prediction for the collapse distances. These demonstrate the consistency of the dynamics of the spatial collapse in a HCF detailed before.

**Table 2:** Group velocity and GVD values for the fundamental and first three excited modes at 1030 nm in a HCF with 150 *μ*m core radius and filled with argon at 1 bar.

*λ* = 1030 nm	*EH*_11_	*EH*_12_	*EH*_13_	*EH*_14_
*v*_*g*_ (nm/fs)	299.918	299.913	299.906	299.894
GVD (*fs*^2^/m)	16.4	−37.3	−133.9	−273.4

**Figure 7:**
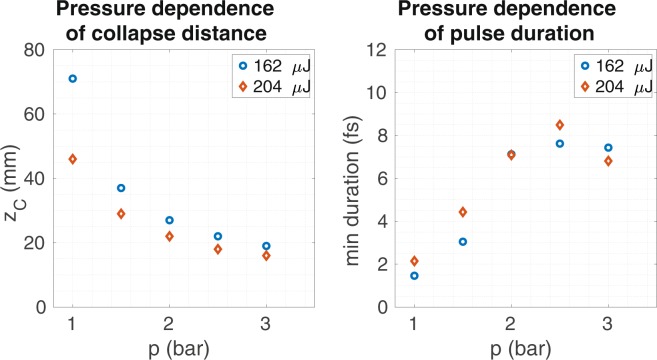
We show the collapse distance (left) and the shortest achieved temporal duration (right) of a 30-fs pulses with fixed input energy (162 *μ*J, which corresponds to *P*_*in*_/${P}_{cr}=0.93$ at 1 bar (blue circles), and 204 *μ*J, which corresponds to *P*_*in*_/${P}_{cr}=1.17$ at 1 bar (orange diamonds)) at 800 nm propagating in the *EH*_11_ mode as a function of the pressure in a HCF with 150 *μ*m core radius, filled with argon.

Gas pressure is another parameter which has an important role in nonlinear propagation in HCFs^7,31^ because it affects both linear dispersion and nonlinear coupling. In order to study the possible use of the pressure as a control parameter to locate the collapse at a desired distance, we have performed a pressure scan for a 30 fs Ti:Sa laser pulse with fixed input energy propagating inside a HCF with core radius 150 *μ*m filled with Ar. The results shown in Fig. 7 (left) demonstrate that gas pressure is indeed an excellent parameter to tune the spatial collapse position. As the pressure increases the critical power decreases so the beam collapses at shorter distances. For gas pressures below 1 bar we do not observe collapse since we enter in the ${P}_{in}\ll {P}_{cr}$ region. Moreover, to have an idea of how the temporal dynamics changes during this pressure scan we show the shortest pulse obtained for each case in Fig. 7 (right). One can observe that the lower the pressure, i.e. the lower the nonlinearity, the more necessary is the temporal self-compression effect to achieve the nonlinear spatial collapse.

### The trace of the self-focusing process in a real experiment: the time-dependent model ((2 + 1)D) including ionization

All the results presented before have been obtained with models which do not include the effects of the generated plasma in the HCF, in order to isolate the spatial dynamics of the self-focusing process. Therefore, one cannot compare directly those results with experiments, where other high nonlinear effects such as the gas ionization eventually prevent the spatial collapse. In this section we will add the gas ionization, the losses due to the ionization process and due to the plasma absorption to the (2 + 1)D model (see Methods) in order to prove that the collapse dynamics shown before is an useful tool to find out the energy limit of a standard HCF post-compression setup.

**Figure 8:**
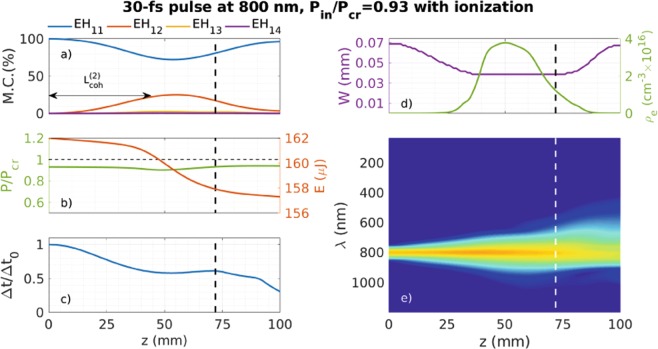
Propagation of a 30 fs pulse at 800 nm with ${P}_{in}=0.93{P}_{cr}$, propagating in a 150 *μ*m core radius HCF filled with 1 bar of Ar including ionization. We show the evolution of the percentage mode contribution (M.C.) of the first four spatial modes (**a**), the evolution of the peak power, indicating the *P*_*cr*_ threshold with an horizontal dashed line, and the pulse energy (**b**), the evolution of the on-axis pulse duration (**c**), the evolution of the spatial width and the plasma density (**d**) and the evolution of the on-axis spectrum (log scale) (**e**) with the propagation distance. A vertical dashed line indicates the collapse distance (72 mm) obtained with the pure self-focusing model (without ionization).

Figure 8 shows the complete dynamics, including the ionization effects, for the same conditions shown in Fig. 4 (right). It is clear that the beam collapse does not occur for this particular input power, although according to the spatial width, the beam still presents clear self-focusing dynamics which is not able to achieve the collapse due to the appearance of the plasma (see the peak plasma density shown in Fig. 8(d)). As a consequence of the arrest of the spatial dynamics, the temporal self-compression slows down, preventing the peak power increase obtained in the pure self-focusing case and, as a consequence, showing a narrower spectral broadening. Although these new simulations bring more nonlinear terms into play, they mutually counteract, making difficult to identify them by simply observing the energy or spatial structure of the output pulse.

**Figure 9:**
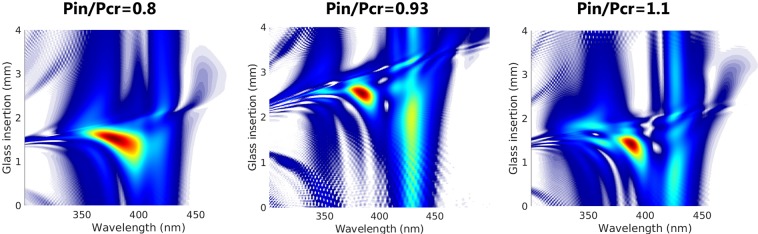
D-scan traces of the output field after the propagation (including ionization) inside a 20 cm long and 150 *μ*m core radius HCF filled with 1 bar of argon of an input 30-fs pulse at 800 nm with an initial *EH*_11_ mode spatial structure. The values of the input powers are *P*_*in*_/${P}_{cr}=0.8$ (left), *P*_*in*_/${P}_{cr}=0.93$ (middle) and *P*_*in*_/${P}_{cr}=1.1$ (right).

The best way to realize that this propagation presents strong nonlinear dynamics is through the phase of the pulse, that retains most of this information. For example, one could use the d-scan^35^, which is a technique to compress and perform a complete measurement of the pulse, including the phase information. It is well-known that the d-scan trace is a perfect tool to optimize a standard HCF post-compression setup^7^. To achieve the optimum stable and shortest output pulse the d-scan trace must present a long and smooth structure, in many cases slightly tilted due to the third order dispersion acquired during the nonlinear propagation. Figure 9 shows the d-scan trace of the output field after the propagation (including ionization) inside a 20 cm long and 150 *μ*m core radius HCF filled with 1 bar of argon of an input 30-fs pulse at 800 nm with an initial *EH*_11_ mode spatial structure. The values of the input powers are *P*_*in*_/${P}_{cr}=0.8$ (left), *P*_*in*_/${P}_{cr}=0.93$ (middle) and *P*_*in*_/${P}_{cr}=1.1$ (right). The two cases with the higher input powers show spatial collapse, according to the pure self-focusing simulation (without ionization), and they also show structured d-scan traces, useless for post-compression applications. Only the *P*_*in*_/${P}_{cr}=0.8$ case, which does not undergo collapse according to the pure self-focusing simulations, shows a nice d-scan trace, close to the optimum^7^. Figure 9 demonstrates that the isolated self-compression study is a solid tool to identify the energy limits of the standard HCF self-compression setup, which are those that do not present spatial collapse.

## Conclusion

We have demonstrated that the confinement of the fundamental spatial mode *EH*_11_ in a HCF plays a key role in the self-focusing process, minimizing the diffraction and enhancing self-focusing. We have identified two different regions related to the self-focusing process in the HCF. For input peak powers slightly greater than *P*_*cr*_ the collapse appears at shorter distances than in the free case and independently of the pulse duration. For input peak powers below *P*_*cr*_ we have obtained spatial collapses induced mainly by the energy transfer process between spatial modes. The interplay between the spatial modes explains the discrete collapse distance regions that we have numerically observed. In this low peak power region the spatial collapse is more complex and depends not only on the peak power but also on the pulse duration. The spatial collapse dynamics explained here can be used to identify the energy limits when up-scaling the standard post-compression schemes based on HCFs.

## Methods

To study the collapse process in HCFs we have developed two numerical models based on the standard nonlinear envelope propagation equation.

### The time-independent model ((1 + 1)D model)

The early numerical studies of self-focusing dynamics solved a propagation equation including the diffraction and self-focusing effects, and neglecting any time-dependent term^24^. To study the spatial dynamics of the laser pulse inside the HCF, we have first used a similar time-independent model ((1 + 1)D model) based on the nonlinear Schrödinger equation (NLSE)^36^:

3 $$\frac{\partial E(r,z)}{\partial z}=(\hat{L}+\hat{N})E(r,z)$$

where *E*(*r*, *z*) is the spatial envelope of the laser beam. Assuming cylindrical symmetry, the two terms that govern the beam propagation, $\hat{L}$ and $\hat{N}$, are written as follows:

4 $$\hat{L}=\frac{i}{2{k}_{0}}(\frac{{\partial }^{2}}{\partial {r}^{2}}+\frac{1}{r}\frac{\partial }{\partial r}+{k}_{0}^{2}({n}_{0}^{2}(r)-1))-\frac{\alpha }{2}$$

5 $$\hat{N}=i{k}_{0}{n}_{0}(r){n}_{2}(r){|E(r,z)|}^{2}$$

The linear part of the propagation equation, $\hat{L}$, represents the diffraction and the absorption of the fundamental mode in the HCF. The nonlinear part, $\hat{N}$, represents the self-focusing process induced by the Optical Kerr effect^37^. ${k}_{0}=2\pi$/*λ*_0_, *λ*_0_ being 800 nm, and *α* is the absorption of the fundamental mode *EH*_11_^38^. In our case we simulate a HCF filled with argon at 1 bar, so ${n}_{0}=1.0003$ and ${n}_{2}=1.74\cdot {10}^{-23}\,{{\rm{m}}}^{2}$/W in the core^39^, and ${n}_{0}=1.45$ and ${n}_{2}=3.2\cdot {10}^{-20}\,{{\rm{m}}}^{2}$/W in the fused silica cladding^18^. For the case of the Yb-based pulses we use a ${n}_{2}=0.93\cdot {10}^{-23}\,{{\rm{m}}}^{2}$/W^29^ for the argon in the core. Note that there are different values of the *n*_2_ parameter in the literature, for instance ${n}_{2}=0.97\cdot {10}^{-23}\,{{\rm{m}}}^{2}$/W for *λ* = 800 nm^40^, but since we present the results as a function of the ratio *P*_*in*_/*P*_*cr*_, they do not depend on the particular value of *n*_2_.

Equation 3 is solved using a standard split-step method, solving the linear operator with a Crank-Nicolson scheme and the nonlinear part is solved as an intensity-dependent phase factor. We use an uniform grid for both coordinates, with transversal and longitudinal step sizes Δ*r* = 2 *μ*m and Δ*z* = 5 *μ*m, respectively. These step sizes fulfill the stability condition Δ*z*/${k}_{0}{({\rm{\Delta }}r)}^{2} < 1$/4, which is a strong restriction on the propagation step size^37^.

### The time-dependent model ((2 + 1)D)

A more complete description of the pulse propagation is simulated with a time-dependent model ((2 + 1)D model) that includes the spatial and temporal dynamics of the pulse in the core of the fiber. The propagation equation for the temporal envelope of the pulse, $E(r,z,T)$, is

6 $$\frac{\partial E(r,z,T)}{\partial z}=(\hat{L}+\hat{N})E(r,z,T)$$

The equation is solved again with a split-step method, the linear part is solved decomposing the input pulse in the *EH*_1*m*_ spatial modes of the HCF, and the nonlinear part is solved by means of a fourth-order Runge-Kutta algorithm in the time domain (see^7^ for more details). We use thirty modes for the modal decomposition, which are enough to model the beam dynamics (we have checked that the energy transferred to the highest modes is almost negligible). The first part of the equation, $\hat{L}$, represents the linear propagation effects that we solve using the complex propagation coefficient of each mode, ${\beta }_{m}(\omega )$.

7 $$A(r,z+{\rm{\Delta }}z,\omega )=\sum _{m=1}^{\infty }\,{c}_{m}(\omega ,z)E{H}_{1m}(r){\exp }(i{\beta }_{m}(\omega ){\rm{\Delta }}z))$$

The nonlinear part, $\hat{N}$, includes self-focusing, self-phase modulation and self-steepening.

8 $$\hat{N}E(r,z,T)=i{k}_{0}{n}_{0}{n}_{2}(1+\frac{i}{{\omega }_{0}}\frac{\partial }{\partial T})({|E(r,z,T)|}^{2}E(r,z,T)),$$

where now *n*_0_ and *n*_2_ represents the linear and nonlinear refractive index of the gas filling the HCF, respectively, and ${\omega }_{0}$ is the central frequency. We use a local time frame, defined as $T=t-z$/*v*_*g*_, being *v*_*g*_ the group velocity of the fundamental spatial mode of the HCF (*EH*_11_).

To better identify the collapse dynamics, gas ionization inside the HCF, that would inhibit it, has not been taken into account, as done in many other self-focusing studies^12,24,25^. Obviously the self-focusing process will eventually activate ionization, changing the spatio-temporal evolution of the beam since then. Such an intense nonlinear propagation introduces a very complex spectral phase that makes the output pulse not useful for post-compression. In other words, we are interested in the dynamics of the approach to blow up instead of in blow up itself^25^.

### The time-dependent model ((2 + 1)D) including ionization

To be able to compare our results with experiments we have also used a time-dependent model ((2 + 1)D model) including, in addition to all the terms explained in the previous model, the ionization and all the losses related to that process. We have added the following two new terms into the nonlinear part of the propagation equation detailed above:

9 $${\hat{N}}_{ioniz}(E(r,z,T))=-\,i\frac{\sigma {\omega }_{0}{\tau }_{C}}{2}{(1+\frac{i}{{\omega }_{0}}\frac{\partial }{\partial T})}^{-1}[\rho (r,T)E(r,z,T)],$$

10 $$\begin{array}{rcl}{\hat{N}}_{abs}(E(r,z,T)) & = & -\,\frac{W(|E{|}^{2}){U}_{i}}{2|E{|}^{2}}({\rho }_{at}-\rho )\\  &  & -\,\frac{\sigma }{2}{(1+\frac{i}{{\omega }_{0}}\frac{\partial }{\partial T})}^{-1}[\rho (r,T)E(r,z,T)],\end{array}$$

where *σ* is the cross section for the inverse Bremsstrahlung calculated as^41^ $\sigma ={k}_{0}{\omega }_{0}{\tau }_{C}$/$({n}_{0}{\rho }_{C}(1+{\omega }_{0}^{2}{\tau }_{C}^{2}))$, ${k}_{0}=2\pi$/*λ*_0_, ${\omega }_{0}$ is the central frequency, *n*_0_ the linear index of refraction of the gas filling the HCF, ${\rho }_{C}=7.403\cdot {10}^{20}\,c{m}^{-3}$ is the critical density, ${\tau }_{C}=350\,{\rm{fs}}$ represents the collision time and $\rho$ is the ionized electron density. The ionization dynamics are obtained from $\partial \rho /\partial T=W(|E{|}^{2})\,({\rho }_{at}-\rho )$, where $W(|E{|}^{2})$ are the PPT ionization rates^42^ and ${\rho }_{at}=2.7\cdot {10}^{19}\cdot p\,c{m}^{-3}$ (being *p* the gas pressure in bar) is the atomic density of the medium. In Eq. 10, that includes the losses due to the ionization process (first term) and the losses due to the plasma absorption (second term), ${U}_{i}=15.76\,{\rm{eV}}$ represents the ionization potencial of the gas.
